# Impact of the malnutrition on mortality in Rheumatoid arthritis patients: A cohort study from NHANES 1999–2014

**DOI:** 10.3389/fnut.2022.993061

**Published:** 2023-01-04

**Authors:** Pan Tian, Jialing Xiong, Wanxia Wu, Shanshan Shi, Aizhen Chen, Kaihong Chen, Weihua Chen, Aiyu Wu, Ying Liao

**Affiliations:** ^1^Department of Rheumatology, Longyan First Affiliated Hospital of Fujian Medical University, Longyan, China; ^2^Department of Cardiology, Longyan First Affiliated Hospital of Fujian Medical University, Longyan, China; ^3^Department of Internal Medicine, The School of Clinical Medicine, Fujian Medical University, Fuzhou, China

**Keywords:** Rheumatoid arthritis, malnutrition, mortality, prognosis, NHANES

## Abstract

**Background:**

Patients with Rheumatoid arthritis (RA) are prone to malnutrition. However, it is rare studies assessing the relationship between malnutrition and all-cause mortality in patients with RA.

**Objective:**

To investigate the relationship between malnutrition and all-cause mortality in patients with RA in a large national sample cohort.

**Methods:**

We analyzed data on 1,976 adults ≥ 18 years of age during National Health and Nutrition Examination Survey (NHANES) 1999–2014. We chose the Controlled Nutritional Status Score (CONUT) and the Nutritional Risk Index (NRI) to assess the nutritional status of patients with RA. The Kaplan–Meier (KM) survival curves Cox proportional hazards regression models were used to analyze the associations between malnutrition and all-cause mortality.

**Results:**

Of the 1,976 patients with RA (57.38 ± 0.40 years, female 59.9%, non-Hispanic white 69.9%), the prevalence of malnutrition was 18.8% by used the CONUT and 26.6% by used the NRI. The KM survival curves showed that malnutrition was associated with a higher incidence of all-cause mortality during the 10-year follow-up period (log-rank test, *P* < 0.001). In the fully corrected model, the adjusting hazard ratio (aHR) for all-cause mortality in patients with moderate to severe malnutrition with CONUT and NRI were 5.63 (95% CI, 2.55–12.45; *P* < 0.001) and 2.56 (95% CI, 1.81–3.62; *P* < 0.001), respectively, compared with patients without malnutrition.

**Conclusion:**

Malnutrition is very prevalent in patients with RA, approximately 18.8% (CONUT) to 26.6% (NRI). Malnutrition is strongly associated with an increased risk of all-cause mortality. These findings underscore the importance of attention and intervention in the nutritional status of patients with RA. Further clinical trials are needed to prospectively assess the effect of nutritional interventions on the prognosis of patients with RA.

## Introduction

Rheumatoid arthritis (RA) is an autoimmune disease characterized by joint inflammation that affects about 1% of the population. It affects at least twice as many women as men, and although it can occur at any age, the highest incidence is at age 50. Uncontrolled active RA has a poor prognosis, leading to joint damage, disability, decreased quality of life, and cardiovascular and other comorbidities (1, 2). In addition, RA imposes a heavy financial burden, with increased hospital stays, sick leave, and work-related disability taking a toll on the economy (3).

Malnutrition has been shown to affect quality of life in chronically ill elderly populations (4). Disease-related malnutrition is strongly associated with morbidity, disability, short- and long-term mortality, and impaired disease recovery (5). Patients with RA are vulnerable to malnutrition (6). Previous study shows that about two-thirds of RA patients have malnutrition (7, 8). The high incidence of malnutrition in RA patients may be related to the inflammatory response during the disease, metabolic abnormalities, and disuse atrophy due to activity restriction (9, 10).

An observational study found that malnutrition itself may be a factor in the increased morbidity and mortality of RA (8). However, its sample size was small and there are no large sample studies assessing the relationship between malnutrition and poor prognosis in patients with RA. Therefore, there is a need to validate it in a large sample representing a national cohort. Our study was to investigate the relationship between malnutrition and all-cause mortality in patients with RA in a large national sample cohort.

## Materials and methods

### Study populations

**FIGURE 1:**
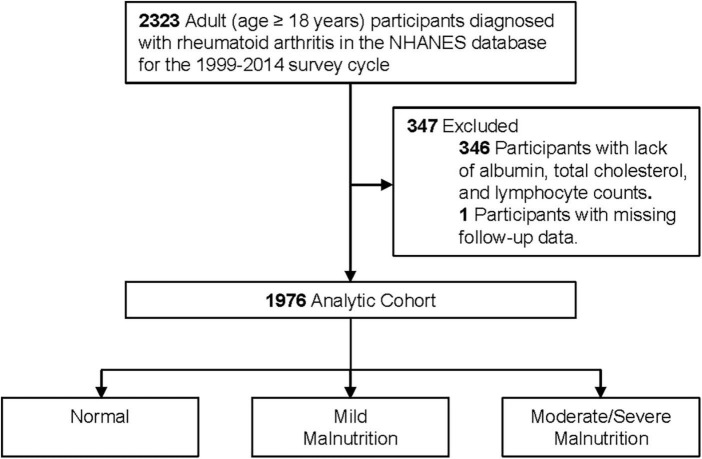
Flow chat.

This was a retrospective cohort study based on patients aged ≥ 18 years with a diagnosis of RA in the National Health and Nutrition Examination Survey (NHANES) database 1999–2014. Among these participants, we excluded patients with lack of albumin, total cholesterol, and lymphocyte counts (*n* = 346) and excluded patients with missing follow-up data (*n* = 1), resulting in the inclusion of 1,976 patients (Figure 1). NHANES is a nationally representative health survey designed and administered by the National Center for Health Statistics (NCHS) at the Centers for Disease Control and Prevention (CDC). The NCHS ethics review board has approved the NHANES protocol. Written informed consent was obtained from each participant.

The presence of RA depends on the subject’s answers to two main questions: “Has a doctor or other health professional ever told you that you had arthritis?” – “Yes.” and “Which type of arthritis was it?”–“Rheumatoid arthritis.” The above questionnaires were completed by professionally trained investigators with strict quality control.

### Malnutrition screening tools

Since the characteristic progression of malnutrition in patients with RA is attributed to excessive protein hydrolysis metabolism and disuse atrophy caused by the inflammatory cytokine (10), which is indexed by albumin values in both the Controlled Nutritional Status Score (CONUT) and the Nutritional Risk Index (NRI), we chose the CONUT and NRI to better assess the nutritional status of patients with RA.

The CONUT was developed by Ulibarri et al. (11). In 2005 as a screening tool for nutritional status in hospitalized patients. It is scored based on albumin, total cholesterol, and lymphocyte count, with a score of 0 to 1 indicating normal; 2 to 4 indicating mild malnutrition, 5 to 8 indicating moderate malnutrition, and 9 to 12 indicating severe malnutrition.

The NRI was a nutritional status score that had become popular in recent years, Buzby et al. originally used the formula 1.519 × serum albumin (g/L) + 41.7 × [current body weight (kg)/usual body weight (kg)] to calculate the NRI (12). The usual body weight was replaced by ideal body weight, according to previous studies (13) and using the Lorenz formulas: height (cm) − 100–[(height (cm) − 150)/4] for male and height (cm) − 100–[(height (cm) − 150)/2.5] for female. When the current weight exceeds the ideal weight, we set the weight as current weight/ideal weight = 1. We classified patients into four nutritional risk categories: severe malnutrition (NRI < 83.5), moderate malnutrition (83.50 ≤ NRI < 97.49), mild malnutrition (97.50 ≤ NRI < 100), and normal (NRI ≥ 100). We combined severe malnutrition with moderate malnutrition because the number of severe malnourished patients was low.

### Outcome

The primary outcome was all-cause mortality. We used data from the NHANES public-use linked mortality file, which was linked by the NCHS to the National Death Index, with follow-up through 31 December 2015. Case definitions for underlying causes of death were according to the *International Classification of Diseases, Tenth Revision (ICD-10)* (14).

### Statistical analysis

The continuous variables were presented as mean [standard error (SE)] or medians [interquartile range (IQRs)], and categorical variables were presented as frequency counts and percentages. Time-to-event data were presented graphically using Kaplan–Meier (KM) curves. Cox proportional hazards regression models were used to estimate hazard ratios (HR), and 95% confidence interval (CI) for the associations between malnutrition and all-cause mortality. Model 1 was a crude model unadjusted for potential confounders. Model 2 was adjusted for demographic factors, including age, sex, race, smoke, and body mass index (BMI). Model 3 was further adjusted for coronary heart disease (CHD), congestive heart failure (CHF), hypertension, cancer, and diabetes mellitus (DM). We stratified patients into subgroups according to age, sex, and BMI and examined the association between malnutrition and all-cause mortality. To test whether the pattern of association varies across stratifications, we estimated multiplicative interactions by including the product term (exposure × stratification variable) in the models. All statistical analyses were performed using R, version 4.0.3 software (R Foundation for Statistical Computing, Vienna, Austria). All *p* values were two sided, and values < 0.05 were considered significant.

## Results

### Clinical characteristics

Of the 1,976 patients with RA, most were female (59.9%), most were non-Hispanic white (69.9%), and the mean age was 57.38 years. The mean albumin value of the study population was 4.18 g/dL, the cholesterol level was 5.13 mmol/L, and the mean lymphocyte value was 2.09 × 10^3^/μL. There were 1,258 (57.5%) RA patients with hypertension, 776 (32.3%) with DM, 171 (8.4%) with CHD, 184 (8.8%) with CHF and 285 (15.8%) with cancer. Additional baseline characteristics data for the study population were detailed in Table 1.

**TABLE 1:** Baseline characteristics of the study population^a^.

Characteristic	Overall (*n* = 1,976)
**Demographic and anthropometric data**	
Age, mean (SD), years	57.38 (56.97–57.78)
Female, *n* (%)	1174 (59.9)
**Race/ethnicity, *n* (%)**	
Hispanic	333 (5.9)
Non-Hispanic white	890 (69.9)
Non-Hispanic black	557 (15.7)
Other	196 (8.5)
Height, mean (SD), cm	166.40 (166.10–166.70)
Weight, mean (SD), kg	83.55 (82.79–84.32)
BMI, mean (SD), kg/m^2^	30.06 (29.82–30.31)
Smoke, *n* (%)	991 (58.7)
Drink, *n* (%)	691 (68.2)
**Laboratory data**	
Triglyceride, mean (SD), mmol/L	1.66 (1.62–1.70)
Cholesterol, mean (SD), mmol/L	5.13 (5.10–5.16)
Albumin, mean (SD), g/dL	4.18 (4.17–4.19)
Neutrophile granulocyte, mean (SD), × 10^3^/μL	4.48 (4.42–4.54)
Lymphocyte, mean (SD), × 10^3^/μL	2.09 (2.06–2.11)
**Comorbidities**	
Hypertension, *n* (%)	1258 (57.5)
Diabetes mellitus, *n* (%)	776 (32.3)
Coronary heart disease, *n* (%)	171 (8.4)
Congestive heart failure, *n* (%)	184 (8.8)
Cancer, *n* (%)	285 (15.8)
**Malnutrition**	
**Continuous, mean (SD),**	
CONUT	0.77 (0.74–0.80)
NRI	103.58 (103.41–103.75)
**Any degree of malnutrition, *n* (%)**	
CONUT	407 (18.8)
NRI	557 (26.6)

SD, standard deviation; BMI, body mass index; CONUT, the Controlled Nutritional Status Score; NRI, the Nutritional Risk Index.

^a^All means and SDs for continuous variables and percentages for categorical variables were weighted, with the exception of the number of participants. Because all numbers were rounded, percentages may not total 100%.

### Prevalence of malnutrition

The prevalence of malnutrition was 18.8% by used the CONUT and 26.6% by used the NRI (Table 1). In Table 2, mild malnutrition was present in 391 (18.3%) and 307 (15.6%) patients by used the CONUT and NRI, respectively, compared with 16 (0.4%) and 250 (11.0%) patients with moderate to severe malnutrition, respectively.

**TABLE 2:** Prevalence of malnutrition according to two different scoring systems.

Nutritional indices		Risk of malnutrition			
		**Absent**	**Mild**	**Moderate**	**Severe**
**CONUT, points**		0–1	2–4	5–8	9–12
Formula	Albumin, g/dL (score)	≥3.5 (0)	3.0–3.4 (2)	2.5–2.9 (4)	<2.5 (6)
	Total cholesterol, mmol/L (score)	≥180 (0)	140–199 (1)	100–139 (2)	<100 (3)
	Lymphocyte count, × 109/l (score)	≥1.60 (0)	1.20–1.59 (1)	0.80–1.19 (2)	<0.80 (3)
Study population, *n* (%)		1569 (81.2)	391 (18.3)	16 (0.4)	0 (0.0)
**NRI, points**		≥100	97.50–99.99	83.50–97.49	<83.50
Formula	1.519 × serum albumin (g/L) + 41.7 × [current body weight [kg]/ideal weight (kg)]				
Study population, *n* (%)		1419 (73.4)	307 (15.6)	246 (10.8)	4 (0.2)

CONUT, the Controlled Nutritional Status Score; NRI, the Nutritional Risk Index.

### Associations between malnutrition and mortality

**FIGURE 2:**
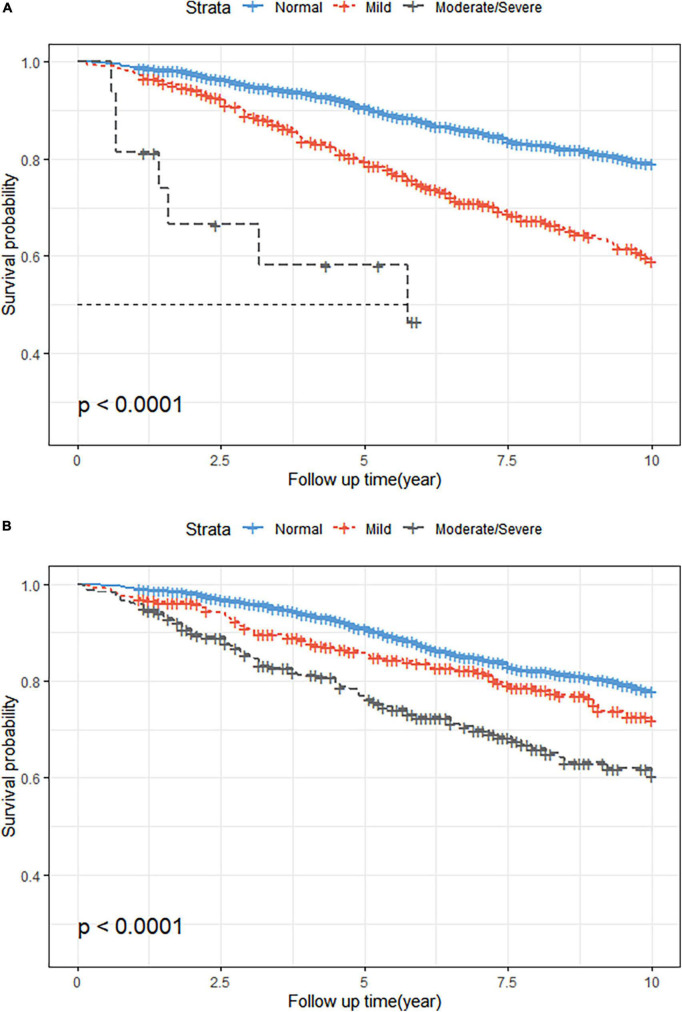
Kaplan-Meier (KM) survival curves stratified by different malnutrition status. **(A)** KM curves according to the Controlled Nutritional Status Score (CONUT) (unweighted). Log-rank test, *P* < 0.001. **(B)** KM curves according to the Nutritional Risk Index (NRI) (unweighted). Log-rank test, *P* < 0.001.

The KM survival curves showed that malnutrition was associated with a higher incidence of all-cause mortality during the 10-year follow-up period, regardless of whether the CONUT or NRI score was used (log-rank test, *P* < 0.001; Figure 2). The Cox proportional risk regression analysis showed that the risk of all-cause mortality increased for each one CONUT score increase (aHR, 1.21; 95% CI, 1.10–1.34; *P* < 0.001) and decreased for each one NRI score increase (aHR, 0.93; 95% CI, 0.91–0.95; *P* < 0.001). In the fully corrected multifactorial model 3, compared with patients with normal nutritional status, all-cause mortality aHR was 1.51 (95% CI, 1.09–2.08; *P* = 0.013) and 2.26 (95% CI, 1.67–3.06; *P* < 0.001) in patients with mild malnutrition diagnosed by CONUT and NRI, respectively. Moderate to severe malnutrition according to CONUT and NRI had an all-cause mortality aHR of 5.63 (95% CI, 2.55–12.45; *P* < 0.001) and 2.56 (95% CI, 1.81–3.62; *P* < 0.001), respectively (Table 3).

**TABLE 3:** All-cause mortality hazard ratios (HRs) for participants aged 18 years and older according to malnutrition status: National Health and Nutrition Examination Survey (NHANES) survey 1999–2014 with follow-up through 2015 (Weighted).

Multivariable status	Model 1		Model 2		Model 3	
	**HR (95%CI)**	***P* value**	**HR (95%CI)**	***P* value**	**HR (95%CI)**	***P* value**
**CONUT, continuous**						
Per 1-score increment	1.37 (1.23–1.51)	<0.001	1.23 (1.12–1.36)	<0.001	1.21 (1.10–1.34)	<0.001
**CONUT, categorical**						
Normal	Ref		Ref		Ref	
Mild	1.96 (1.46–2.62)	<0.001	1.49 (1.10–2.01)	0.010	1.51 (1.09–2.08)	0.013
Moderate/severe	9.97 (4.25–23.37)	<0.001	7.27 (2.91–18.12)	<0.001	5.63 (2.55–12.45)	<0.001
**NRI, continuous**						
Per 1-score increment	0.94 (0.92–0.96)	<0.001	0.93 (0.91–0.95)	<0.001	0.93 (0.91–0.95)	<0.001
**NRI, categorical**						
Normal	Ref		Ref		Ref	
Mild	1.59 (1.18–2.14)	0.003	2.17 (1.58–2.97)	<0.001	2.26 (1.67–3.06)	<0.001
Moderate/severe	2.39 (1.70–3.34)	<0.001	2.59 (1.81–3.70)	<0.001	2.56 (1.81–3.62)	<0.001

CONUT, the Controlled Nutritional Status Score; NRI, the Nutritional Risk Index.

Model 1: No adjusted.

Model 2: Adjusted by age, sex, race, smoke, body mass index.

Model 3: Adjusted by age, sex, race, smoke, body mass index, coronary heart disease, congestive heart failure, hypertension, cancer, diabetes mellitus.

### Subgroups analyses

**FIGURE 3:**
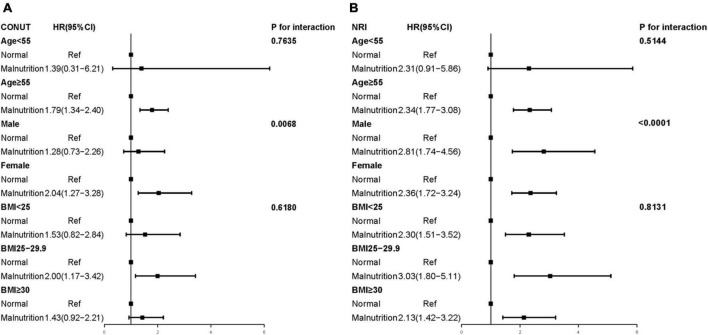
Multivariate Cox proportional hazards regression models of different malnutrition status and all-cause mortality (Weighted). **(A)** CONUT, the Controlled Nutritional Status Score; **(B)** NRI, the Nutritional Risk Index; BMI, body mass index. Adjusted by age, sex, race, smoke, body mass index, coronary heart disease, congestive heart failure, hypertension, cancer, and diabetes mellitus.

In the subgroup analysis of CONUT score, the malnourished female population (aHR, 2.04; 95% CI, 1.27–3.28; *P* = 0.003) had a higher risk of all-cause mortality than the male population (aHR, 1.28; 95% CI, 0.73–2.26; *P* = 0.394). Conversely, in the subgroup analysis of NRI score, the malnourished male population (aHR, 2.81; 95% CI, 1.74–4.56; *P* < 0.001) had a higher risk of all-cause mortality than the female population (aHR, 2.36; 95% CI, 1.72–3.24; *P* < 0.001). In the CONUT and NRI scores, we could observe a significant interaction between malnutrition and sex (CONUT: *P*-interaction = 0.0068; NRI: *P*-interaction < 0.0001; Figure 3), while there was no interaction in the age and BMI subgroups (all *P*-interaction > 0.05).

## Discussion

In this retrospective study, the prevalence of malnutrition was higher in the RA population according to CONUT and NRI scores. Our study showed that the risk of all-cause mortality was higher in the malnourished RA population compared to the RA population with normal nutritional status. Therefore, there is a need to screen patients with RA for malnutrition.

RA is a chronic systemic inflammatory disease that, can lead to joint damage, disability, and decreased quality of life, as well as cardiovascular disease and other comorbidities (2). Moreover, RA imposes a heavy financial burden on patients (3). The prevalence of malnutrition in RA patients in previous relevant studies is high, which prevalence of malnutrition was more than two-thirds (7, 8). The high incidence of malnutrition in RA patients may be attributed to several factors. It is a systemic inflammatory disease, and inflammation drives malnutrition during the disease, often with metabolic effects, including insulin resistance and decreased appetite (9). Excessive proteolytic metabolism and disuse atrophy due to functional impairment caused by inflammatory cytokines also contribute to the high prevalence of malnutrition in rheumatoid off patients (10). Based on previous theoretical foundations, we chose CONUT and NRI scores to comprehensively assess the nutritional status of patients with RA and to further investigate the impact of the degree of malnutrition on the poor prognosis of patients with RA. Similar to that observed in a small sample observational study by R Collins Jr et al. (8) our study found that patients with poorer nutritional status had a worse prognosis. It is noteworthy that we used two different objective nutrition scoring tools and validated them in a large national sample.

In the subgroup results, we found several special features. First, in the age subgroup results, we found that the prognosis of RA patients aged ≥ 55 years with malnutrition was worse than that of patients aged < 55 years. With the coexistence of multiple diseases and progressive aging of physiological functions and tissues and organs in the elderly, the blow of malnutrition seems to be more fatal. RA can occur at any age, but the peak incidence is at age 50 years (1). The high incidence of RA and high mortality from malnutrition in this age group are a warning: it is important to assess the extent of malnutrition in elderly RA patients and to improve it by means. Second, RA is very common in females, and in the sex subgroup, females with RA who were diagnosed as malnourished according to CONUT had a worse prognosis than males with RA who were malnourished. Conversely, males with RA who were diagnosed as malnourished according to NRI had a worse prognosis than females. Epidemiological and immunological evidence suggests that female hormones may play a role in the etiology and course of autoimmune diseases such as RA (15). Sex hormone homeostasis is a key factor in regulating immune and inflammatory responses, (16) and it has been proposed that lowering immunosuppressive androgen levels may have a pathogenic role (17). Since the mean year of this study cohort was 57.38 years, the withdrawal of hormone levels in perimenopausal or menopausal females may have deprived females of hormonal protection in the short term and accelerated the progression of RA. Therefore, the prognostic risk due to malnutrition may be masked by the hormonal effects in females, resulting in a lower HR in malnourished female RA patients than in males under NRI criteria. However, studies have shown racial and sex differences in cholesterol levels, with females tending to have higher cholesterol levels than males (18–20). Thus, females diagnosed with malnutrition under CONUT appear to have a worse nutritional status than males, especially having much lower than normal total cholesterol levels. This may be the reason for the higher HR for malnutrition in females than in males under the CONUT criteria. Thirdly, many studies have shown that BMI is strongly associated with all-cause mortality, (21) with the lowest risk of death likely occurring in the range 21–25 kg/m^2^ (22). In the BMI subgroup, RA patients who were overweight (BMI 25–29.9 kg/m^2^) with malnutrition had a higher risk of death than RA patients who were non-overweight (BMI < 25 kg/m^2^) or obese (BMI ≥ 30 kg/m^2^) with malnutrition. Previous study has suggested that obesity is associated with higher survival when high inflammation is combined with malnutrition (23). It has also been suggested that increased BMI is strongly associated with poor prognosis in patients with RA (24, 25). Our data show that the risk of death associated with malnutrition is greater in overweight RA patients than in obese patients. This may be due to the high mortality rate in obese RA patients either with or without malnutrition, resulting in a failure to show the dangers of malnutrition. In addition, as fewer outcome events occurred in the subgroup of BMI < 18.5 kg/m^2^, we analyzed all patients with BMI < 25 kg/m^2^ together. This may have led to a high mortality rate in underweight (BMI < 18.5 kg/m^2^) patients affecting the results. However, malnutrition was independently associated with a high risk of mortality in RA patients, regardless of BMI subgroup.

Our findings strongly suggest that clinicians pay more attention to the nutritional status of patients with RA. Screening for malnutrition in community patients with RA to identify patients at high risk for poor prognosis and providing targeted nutritional interventions and enhanced nutritional management for these patients may improve prognosis. Clinicians should provide scientifically effective nutritional guidance for these high-risk patients based on clinical guidelines, involving oral nutritional supplements, food/fluid fortification or enrichment, dietary counseling, and educational interventions (26). Nutritional interventions and management should begin during hospitalization and continue after discharge to ensure normalization of nutritional status (13). In addition further studies are necessary to explore the effectiveness of different interventions for malnutrition in patients with RA.

However, this study also has several limitations. First, this was a retrospective study using data from the NHANES database from 1999–2014, which may have poor timeliness. Second, the objectivity and accuracy of the nutritional status assessed by the CONUT score and NRI score may be biased due to the lack of comparison with complex comprehensive nutritional screening tools. Thirdly, we did not investigate changes in nutritional status over time and their relationship with outcomes. Fourth, we identified patients with RA based on verbal reports from participants, potentially overestimating actual disease status and underestimating the correlation between patient prognosis and malnutrition in RA. Finally, the results of the BMI subgroup in the subgroup analysis do not reflect the impact of specific body composition on the prognosis of malnourished and RA patients, and underweight and normal weight patients were not well differentiated, pending further research by more scholars.

## Conclusion

Malnutrition is very prevalent in patients with RA, approximately 18.8% (CONUT) to 26.6% (NRI). Malnutrition is strongly associated with an increased risk of all-cause mortality. Malnutrition assessment allows clinicians to determine the high risk of mortality in patients with RA. Adequate assessment of nutritional status and necessary nutritional guidance can help improve the prognosis of patients with RA. Further clinical trials are needed to prospectively assess the effect of nutritional interventions on the prognosis of patients with RA.

## Data availability statement

The datasets presented in this study can be found in online repositories. The names of the repository/repositories and accession number(s) can be found below: The datasets supporting the conclusions of this article are available in the National Health and Nutrition Examination Survey repository (https://www.cdc.gov/nchs/nhanes/).

## Ethics statement

The studies involving human participants were reviewed and approved by The National Center for Health Statistics ethics review board. The patients/participants provided their written informed consent to participate in this study.

## Author contributions

SS: has full access to all the data in the study and takes responsibility for the integrity of the data and the accuracy of the data analysis. PT, SS, AC, WC, and AW: study concept and design. JX, WW, SS, and WC: drafting of the manuscript. PT and SS: statistical analysis. KC: obtained funding. YL: administrative, technical, or material support. YL, KC, and AW: study supervision. All authors: acquisition, analysis, or interpretation of data, critical revision of the manuscript for important intellectual content, contributed to the article, and approved the submitted version.
